# Increased DNA Damage in Progression Of COPD: A Response By Poly(ADP-Ribose) Polymerase-1

**DOI:** 10.1371/journal.pone.0070333

**Published:** 2013-07-24

**Authors:** Ingrid Oit-Wiscombe, Laszlo Virag, Ursel Soomets, Alan Altraja

**Affiliations:** 1 Department of Pulmonary Medicine, Faculty of Medicine, University of Tartu, Tartu, Estonia; 2 Department of Medical Chemistry, Medical and Health Science Centre, University of Debrecen, Debrecen, Hungary; 3 Department of Biochemistry, Faculty of Medicine, University of Tartu, The Centre of Excellence of Translational Medicine, Tartu, Estonia; National Institutes of Health, United States of America

## Abstract

Chronic oxidative stress (OS), a major mechanism of chronic obstructive pulmonary disease (COPD), may cause significant damage to DNA. Poly(ADP-ribose) polymerase (PARP)-1 is rapidly activated by OS-induced DNA lesions. However, the degree of DNA damage along with the evolution of COPD is unclear. In peripheral blood mononuclear cells of non-smoking individuals, non-obstructive smokers, patients with COPD of all stages and those with COPD exacerbation, we evaluated DNA damage, PARP activity and PARP-1 mRNA expression using Comet Assay IV, biotinylated-NAD incorporation assay and qRT-PCR, respectively and subjected results to ordinal logistic regression modelling. Adjusted for demographics, smoking-related parameters and lung function, novel comet parameters, tail length/cell length ratio and tail migration/cell length ratio, showed the greatest increase along the study groups corresponding to the evolution of COPD [odds ratio (OR) 7.88, 95% CI 4.26–14.57; p<0.001 and OR 3.91, 95% CI 2.69–5.66; p<0.001, respectively]. Analogously, PARP activity increased significantly over the groups (OR = 1.01; 95%; p<0.001). An antioxidant tetrapeptide UPF17 significantly reduced the PARP-1 mRNA expression in COPD, compared to that in non-obstructive individuals (p = 0.040). Tail length/cell length and tail migration/cell length ratios provide novel progression-sensitive tools for assessment of DNA damage. However, it remains to be elucidated whether inhibition of an elevated PARP-1 activity has a safe enough potential to break the vicious cycle of the development and progression of COPD.

## Introduction

Of the major mechanisms of chronic obstructive pulmonary disease (COPD), oxidative stress (OS) resulting from direct effects of smoking, but also from persistent inflammation, causes oxidative damage to important biomolecules, including DNA [1]–[3]. Spill-over of the OS to the circulation is associated with a significant systemic disease in COPD [4], [5]. The resulting fall in antioxidant capacity of blood cells could therefore be regarded as a measure of OS in COPD.

Systemic inﬂammation and OS suggest a role for the nuclear enzyme poly(ADP-ribose) polymerase (PARP)-1 (E.C.2.4.2.30) in the pathophysiology of COPD [6]. PARP-1, a member of the PARP superfamily of 18 enzymes, is responsible for more than 90% of the cellular poly(ADP-ribosyl)ation capacity [6]. PARP-1 is activated by reactive oxygen species (ROS)-induced DNA strand breaks, upon which it forms extensive poly(ADP-ribose) polymers from its substrate NAD^+^ [7]. In extensive DNA damage, PARP-1 activation causes depletion of NAD^+^ leading to cell death via reduced glycolysis and mitochondrial respiration [8]. Unlike that on the systemic effects of COPD in general, the data on DNA damage and expression of PARP-1 are inconsistent.

Animal models have shown that PARP-1 activation contributes significantly to the pathophysiology of chronic inflammatory diseases like asthma [9]–[11], diabetes [8], rheumatoid arthritis [12] and chronic colitis [13]. An elevation of PARP-1 in murine hippocampus could serve as proof of PARP-1 as a marker of systemic inflammation [14]. It has been shown, that PARP-1 activity is increased in peripheral blood lymphocytes in patients with COPD [7]. However, we currently tested the hypothesis that the expression of PARP-1 and PARP activity in human peripheral blood mononuclear cells (PBMC) are related to the progression of COPD. In parallel, we employed and developed further comet assay as a sensitive method for detection of DNA strand breaks, alkali-labile sites and delayed repair sites [15].

As far COPD is accompanied by systemic OS, the effect of novel tetrapeptide analogue of glutathione (GSH), UPF17 (4-methoxy-L-tyrosinyl-α-L-glutamyl-L-cysteinyl-glycine) on the PARP-1 activity through changing mRNA expression of PARP-1 was measured.

## Subjects and Methods

### Study Subjects

All participants, recruited from the Department of Pulmonary Medicine, Tartu University Hospital, Tartu, Estonia, were selected to create seven study groups: 1) non-obstructive never-smoking control individuals with normal lung function, 2) current smokers or ex-smokers with normal lung function matching the COPD patients for their demographics and smoking history and habits, 3) four patients groups formed by the four stages of stabile COPD (I-IV) according to the Global Initiative for COPD (GOLD) Guidelines, based on the post-bronchodilator forced expiratory volume in one second (FEV_1_) and 4) patients with a COPD exacerbation according to the GOLD criteria requiring hospitalisation (table 1) [16]. All patients with COPD had their post-bronchodilator FEV_1_/forced vital capacity ratio <0.7 and all showed <12% improvement in FEV_1_ compared with the pre-bronchodilator value. A current smoker was defined as a person, who currently smoked at least one cigarette per day, whereas an ex-smoker was defined as a person, who had quitted smoking for ≥6 month prior to the study. The exclusion criteria included pneumonia, bronchiectasis and any malignancy and all subjects except for those with COPD exacerbation had to be free of viral respiratory infections within the last 2 months. Patients were treated according to the severity of the disease and in accordance with the GOLD guidelines. The study was approved by the Ethics Committee on Human Research of the University of Tartu. Written informed consent was obtained from all participants.

**Table 1:** Characteristics of the individuals involved in the measurement of DNA damage by Comet Assay IV and poly(ADP-ribose) polymerase (PARP) activity in peripheral blood mononuclear cells.

Characteristic	Non-smoking controls (n = 7)	Non-obstructive smokers (n = 4)	Mild COPD(n = 3)	Moderate COPD (n = 7)	Severe COPD(n = 5)	Very severeCOPD (n = 4)	COPD exacerbation (n = 6)	p-value^*^
Age	63.3±2.4	54.7±5.5	62.1±9.9	60.6±3.0	67.7±5.9	59.8±5.5	73.4±3.0	0.16
Male	7 (100.0%)	4 (100.0%)	3 (100.0%)	6 (85.7%)	5 (100.0%)	4 (100.0%)	5 (83.3%)	0.71
BMI	32.2±2.1	28.4±2.7	24.9±1.3	24.9±1.9	23.7±1.4	29.7±2.1	22.6±0.8	0.017
Smoking (pack-years)	-	28.4±9.3	30.7±4.7	34.2±6.0	30.8±5.5	30.1±8.9	53.5±13.6	0.64
Current smoker	-	4 (100.0%)	2 (66.7%)	6 (85.7%)	0 (0%)	2 (50.0%)	4 (66.7%)	0.027
Ex-smoker	-	0 (0.0%)	1 (33.3%)	1 (14.3%)	5 (100.0%)	2 (50.0%)	2 (33.3%)	0.027
FEV_1_, % of predicted	95.9±4.7	99.5±7.0	84.0±2.5	68.7±3.1	35.0±3.7	24.8±2.5	30.2±5.6	<0.001

Data are presented as mean ± SEM or n (%). ^*^To test the equality of the data across the study groups, Kruskal-Wallis test and Pearson’s chi-square test was applied for numeric and nominal variables, respectively; in case of smoking-related variables, non-smoking controls were omitted from the analysis.

### Mononuclear Cell Extraction from Peripheral Blood

PBMC were separated in BD Vacutainer CPT tubes (Becton Dickinson, Franklin Lakes, NJ, USA), by centrifugation at 1500×g for 21 min at 20°C. Isolated PBMC were washed twice with phosphate-buffered saline (PBS). 200 µl of PBMC suspension (6×10^6^ cells/ml) were frozen down in 10% dimethyl sulfoxide (DMSO) and RPMI-1640 [including 10% foetal calf serum (FCS) and 1% penicillin/streptomycin] for the comet assay and measurement of PARP activity. The remaining PBMC were cultured in RPMI-1640 medium for the PARP-1 mRNA expression studies.

### Comet Assay

PBMC were embedded with 0.5% low melting point agarose (Gibco by Life Technologies, Carlsbad, CA, USA) in distilled water and the mixture was added to a microscope slide, pre-coated with 1% of normal melting point agarose in distilled water, and covered with a coverslip. The slide was placed briefly to 4°C for the agarose to achieve solidify. The slides were then immersed in lysis solution [2.5 M NaCl, 100 mM ethylenediaminetetraacetic acid (EDTA) and 10 mM Tris, pH 10.0] containing freshly added 1% triton X-100 and 10% DMSO for at least 1 h at 4°C. Subsequently, the slides were incubated in freshly prepared alkaline buffer (300 mM NaOH and 1 mM EDTA, pH>13) for 40 min for DNA unwinding and electrophoresed in the same buffer. The conditions for electrophoresis were 30 min at 300 mA and 25 V. Following electrophoresis, slides were neutralized with PBS, washed in distilled water and 70% ethanol and dried overnight. The gels were stained for DNA with 1 µg/ml 4′,6-diamidino-2-phenylindole dihydrochloride solution in distilled water and air-dried. Images of 100 randomly selected cells (50 cells from each of two replicate slides) were taken with Zeiss Axiolab microscope (Carl Zeiss, Oberkochen, Germany) and analysed using Comet Assay IV software (Perceptive Instruments Ltd., Suffolk, UK). All standard parameters originally provided by the Comet Assay IV were used. In addition, a set of novel parameters were tested for their ability to more precisely characterise the DNA damage by the progression of COPD: tail moment length, extent tail moment, cell length, tail length/cell length ratio and tail migration/cell length ratio. Tail moment length shows the distance from the centre of the head to the centre of the tail, extent tail moment was calculated by dividing DNA % in the tail by the tail length and cell length shows the distance from the beginning of the head until the end of the tail.

### Detection of PARP Activity

PARP activity was measured using 6-biotin-17-nicotinamide-adenine-dinucleotide (bio-NAD^+^), as previously described [17]. PBMC were cultured on poly-L-lysine-coated coverslips in RPMI medium, supplemented with 10% FCS. After 20 min incubation allowing cells to attach to slides, the medium was removed and replaced with PARP reaction buffer [56 mM 4-(2-hydroxyethyl)-1-piperazineethanesulfonic acid, 28 mM KCl, 28 mM NaCl, 2 mM MgCl_2_, pH 8.0, complemented with 0.01% digitonin and 250 µM biotinylated NAD^+^ immediately before use]. After 60-min incubation at 37°C, the cells were fixed at -20°C in 95% ethanol followed by 10% trichloroacetic acid. The coverslips were rinsed in PBS and endogenous peroxidase was blocked by 0.5% H_2_O_2_/methanol for 15 min. After rinses with PBS, the coverslips were blocked in 1% bovine serum albumin/PBS for 30 min, followed by rinses in PBS–Triton X-100 (0.1%). Incorporated biotin was detected by streptavidin–peroxidase (diluted 1∶100 in PBS–Triton X-100) for 30 min. Coverslips were washed with PBS–Triton X-100 and colour was developed with cobalt-enhanced nickel-3,3′-diaminobenzidine substrate. The coverslips were mounted with glycerol on slides and viewed under a Zeiss Axiolab microscope (Carl Zeiss). The pictures were taken with a Zeiss Axiocam digital camera (Carl Zeiss) and analysed using AlphaView Software (ProteinSimple, Santa Clara, CA, USA).

### Measurement of PARP-1 mRNA Expression

The PBMC suspension was divided into halves with one part cultured in the presence of 500 nM UPF17 and the other without UPF17 that served as a control in RPMI-1640 medium (includes 10% FCS and 1% penicillin/streptomycin). After 12 hours, RNA was extracted from PBMC with the Trizol method according to manufacturer’s protocol (Invitrogen by Life Technologies) and stored at –80°C until cDNA synthesis. cDNA was synthesized with reverse transcriptase reaction, from total RNA (250 ng) using the SuperScript III enzyme (Invitrogen by Life Technologies), according to manufacturer’s instructions.

The gene expression levels were detected using the TaqMan-qRT-PCR method (ABI Prism 7900HT Sequence Detection System, Applied Biosystems by Life Technologies) using Hs00242302_m1 for PARP-1 and hypoxanthine phosphoribosyl-transferase-1 (HPRT-1) as house keeper gene (both from Applied Biosystems by Life Technologies). All reactions were carried out in quadruplicates. The comparative Ct method (ΔCt value) was used for quantification of mRNA, where the amount of target transcript was normalized to the level of endogenous HPRT-1.

### Statistical Analysis

The equality of the baseline data across the study groups was evaluated with Kruskal-Wallis test in numeric variables and Pearson’s chi-square test in nominal variables. Associations of the comet assay parameters, as well as the PARP-1 expression and PARP activity with the presumably increasing degree of impairment from healthy never-smokers through non-obstructive smokers, stable COPD patients of GOLD stages I-IV to patients with COPD exacerbation, were assessed with ordinal logistic regression analysis with calculating the odds ratios (OR) and their 95% confidence intervals (95% CI). Because of the very high collinearity between the comet assay indices due to their derivation, the comet parameters were not simultaneously included into the multivariate model. Instead, the ability of each of the comet assay parameters to indicate the increasing degree of DNA impairment throughout the study groups was assessed separately, though each of them was adjusted for age, gender, body mass index (BMI), lung function (FEV_1%_ predicted), pack-years of smoking and status of a current smoker. The analyses of PARP-1 expression and PARP activity were adjusted similarly.Mann-Whitney U-Test was performed to determine the difference between the COPD patients and non-COPD individuals for the effect of UPF17 on PARP-1 mRNA expression level. p-values below 0.05 were considered to designate statistical significance. All statistical analyses were performed with the Statistical Package for Social Sciences (SPSS, version 17.0, SPSS Inc., Chicago, IL, USA) software.

## Results

### Study Subjects

Different cohorts were used to measure the DNA damage by comet assay and PARP activity (table 1) and the PARP-1 mRNA expression level (table 2). Amongst the patients, who were chosen for DNA damage and PARP activity analysis, there was a difference for smoking history because one patient with COPD exacerbation had a smoking history of 116 pack-years.

**Table 2:** Characteristics of the individuals involved in the measurement of poly(ADP-ribose) polymerase (PARP)-1 mRNA expression in peripheral blood mononuclear cells.

Characteristic	Non-smoking controls (n = 10)	Non-obstructive smokers (n = 12)	Mild COPD(n = 4)	Moderate COPD (n = 14)	Severe COPD (n = 24)	Very severeCOPD (n = 21)	COPD exacerbation (n = 22)	p-value^*^
Age	64.0±3.4	61.1±3.3	76.8±3.0	67.6±2.4	68.8±2.5	70.6±2.5	70.0±2.3	0.098
Male	7 (70.0%)	7 (58.3%)	4 (100.0%)	13 (92.9%)	22 (91.7%)	21 (100.0%)	20 (90.9%)	0.009
BMI	25.7±1.6	27.4±2.0	23.4±1.7	24.3±1.4	25.9±0.9	23.3±1.2	23.4±0.9	0.17
Smoking (pack-years)	-	34.4±10.4	56.3±20.5	42.4±15.5	39.2±22.9	39.4±22.6	43.1±23.5	0.37
Current smoker	-	7 (58.3%)	2 (50.0%)	9 (64.3%)	11 (45.8%)	8 (38.1%)	9 (40.9%)	0.65
Ex-smoker	-	5 (41.7%)	2 (50.0%)	5 (35.7%)	13 (54.1%)	12 (57.1%)	13 (59.1%)	0.75
FEV_1_, % of predicted	97.6±4.5	81.0±5.6	94.5±9.0	60.8±2.6	39.0±1.5	24.2±1.0	33.1±2.9	<0.001

Data are presented as mean ± SEM or n (%). ^*^To test the equality of the data across the study groups, Kruskal-Wallis test and Pearson’s chi-square test was applied for numeric and nominal variables, respectively; in case of smoking-related variables, non-smoking controls were omitted from the analysis.

### DNA Damage, PARP Activity and Expression of PARP-1 mRNA in PBMC

**Figure 1:**
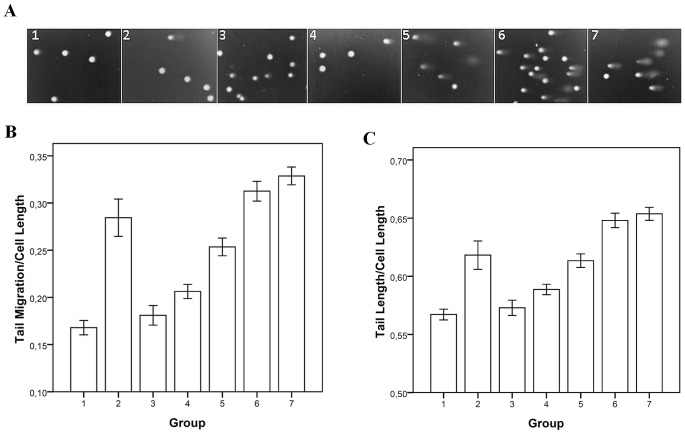
The level of damage to DNA in peripheral blood mononuclear cells. a) Evaluation by comet assay. b) Tail migration/cell length ratio measured with Comet Assay IV. c) Tail length/cell length ratio measured with Comet Assay IV. The study groups are designated as follows: (1) non-smoking, non-obstructive control individuals (n = 7); (2) non-obstructive smokers (n = 4); (3) patients with mild chronic obstructive pulmonary disease (COPD) (n = 3); (4) patients with moderate COPD (n = 7); (5) patients with severe COPD (n = 5); (6) patients with very severe COPD (n = 4); (7) patients with COPD exacerbation requiring hospitalisation (n = 6). Presented as mean ± SEM.

After adjustment for age, gender, BMI, smoking history (pack-years), status of a current smoker and FEV_1%_ predicted, of the Comet IV standard parameters, olive tail moment, DNA % in the tail, head length, tail length and tail migration increased significantly (OR>1.0, p<0.05), whereas DNA % in the head, as well as mean grey level and total intensity decreased significantly (OR<1.0, p<0.05) along the order from healthy control individuals through non-obstructive smokers, patients with increasing stage of stable COPD to those with exacerbation of COPD (table 3, figure 1). Of the derived parameters, tail moment length, extent tail moment and cell length, as well as the novel parameters tail length to cell length ratio and tail migration to cell length ratio also increased significantly to designate escalation to the next group in our study (OR>1.0, p<0.05), (table 3, figure 1). In particular, the comet parameters from PBMC that had the highest OR to reflect the DNA changes along with the evolution of COPD turned out to be the novel parameters tail length/cell length and tail migration/cell length ratios. One unit increase in tail length/cell length ratio and tail migration/cell length ratio was associated with a 7.88-fold (95% CI 4.26–14.57, p<0.001) and a 3.91-fold (95% CI 2.69–5.66, p<0.001) higher probability, respectively, of progressing into the next group of individuals (figure 1, table 3).

**Table 3:** Changing DNA damage in peripheral blood mononuclear cells by Comet Assay IV parameters from never-smoking control individuals through current smokers or ex-smokers with normal lung function and patients with stabile chronic obstructive pulmonary disease (COPD) of the Global Initiative for Chronic Obstructive Lung Disease (GOLD) stages I-IV to patients with a COPD exacerbation, evaluated with ordinal logistic regression analysis.

	Crude		Adjusted*	
Parameter	OR (95% CI)	p-value	OR (95% CI)	p-value
Standard Comet Assay IV parameters				
Olive tail moment	1.093 (1.074–1.112)	<0.001	1.047 (1.025–1.069)	<0.001
Head DNA %	0.985 (0.982–0.988)	<0.001	0.992 (0.988–0.996)	<0.001
Tail DNA %	1.018 (1.015–1.021)	<0.001	1.008 (1.003–1.012)	<0.001
Head length	1.010 (1.002–1.018)	0.019	1.017 (1.005–1.029)	<0.001
Tail length	1.053 (1.047–1.059)	<0.001	1.039 (1.030–1.047)	<0.001
Tail migration	1.050 (1.044–1.056)	<0.001	1.034 (1.026–1.042)	<0.001
Mean grey level	1.013 (1.010–1.017)	<0.001	0.995 (0.990–<1.000)	0.047
Total area	1.0002 (<1.000–1.0005)	0.051	1.0003 (0.9999–1.0006)	0.14
Total intensity	<1.000 (<1.000–>1.000)	0.53	<1.000 (<1.000–<1.000)	<0.001
Width	1.001 (0.993–1.010)	0.75	1.009 (0.997–1.021)	0.15
Derived parameters				
Tail moment length	1.096 (1.084–1.108)	<0.001	1.079 (1.063–1.096)	<0.001
Extent tail moment	1.0005 (1.0004–>1.0006)	<0.001	1.0003 (1.0002–1.0004)	<0.001
Cell length	1.047 (1.041–1.052)	<0.001	1.039 (1.031–1.047)	<0.001
Tail length/cell length^#^	22.459 (14.171–35.595)	<0.001	7.880 (4.264–14.565)	<0.001
Tail migration/cell length^#^	7.583 (5.744–10.012)	<0.001	3.905 (2.694–5.661)	<0.001

* Adjusted for age, gender, body mass index, smoking history (pack-years), status of a current smoker and forced expiratory volume in one second % predicted. ^#^Novel derived parameters.

**Figure 2:**
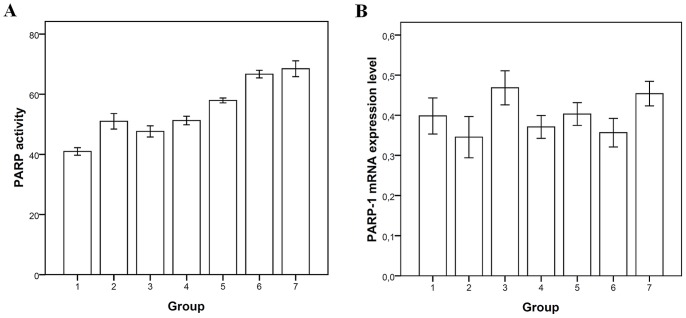
Poly(ADP-ribose) polymerase (PARP) activity and PARP-1 mRNA expression in peripheral blood mononuclear cells. a) PARP activity using biotinylated-NAD incorporation assay. b) PARP-1 mRNA expression levels measured with qRT-PCR. The study groups are designated as follows: (1) non-smoking, non-obstructive control individuals (n = 7 and n = 10 for patients involved in PARP activity and PARP-1 mRNA expression analysis, respectively); (2) non-obstructive smokers (n = 4 and n = 12); (3) patients with mild chronic obstructive pulmonary disease (COPD) (n = 3 and n = 4); (4) patients with moderate COPD (n = 7 and n = 14); (5) patients with severe COPD (n = 5 and n = 24); (6) patients with very severe COPD (n = 4 and n = 21); (7) patients with COPD exacerbation requiring hospitalisation (n = 6 and n = 22). Presented as mean ± SEM.

PARP activity was a significant factor associated with escalation into the next group in our study (OR 1.014, 95% CI 1.006–1.023, p<0.001) (table 4, figure 2). Unlike PARP activity, PARP-1 mRNA expression level did not show an ordinal change accordingly to the groups in our study (table 4, figure 2).

**Table 4:** Changing poly(ADP-ribose) polymerase (PARP) activity and PARP-1 mRNA expression in peripheral blood mononuclear cells from never-smoking control individuals through current smokers or ex-smokers with normal lung function and patients with stabile chronic obstructive pulmonary disease (COPD) of the Global Initiative for Chronic Obstructive Lung Disease (GOLD) stages I-IV to patients with a COPD exacerbation, evaluated with ordinal logistic regression analysis.

	Crude		Adjusted*	
Parameter	OR (95% CI)	p-value	OR (95% CI)	p-value
PARP activity	1.049 (1.043–1.056)	<0.001	1.014 (1.006–1.023)	<0.001
PARP-1 mRNA expression	4.510 (0.448–45.365)	0.20	4.735 (0.378–59.298)	0.23

* Adjusted for age, gender, body mass index, smoking history (pack-years), status of a current smoker and forced expiratory volume in one second % predicted.

### Effect of UPF17 on the Expression of PARP-1 mRNA

**Figure 3:**
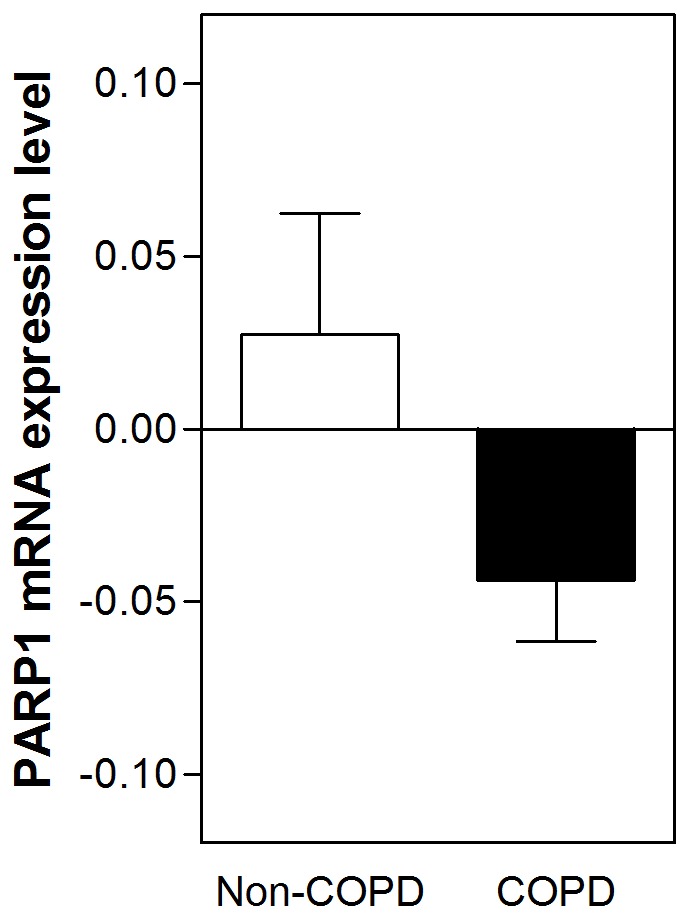
Mann-Whitney U-Test showing a significant down-regulation of the poly(ADP-ribose) polymerase-1 mRNA expression in peripheral blood mononuclear cells from patients with chronic obstructive pulmonary disease (n = 85) by an antioxidant tetrapeptide UPF17 in comparison with that in non-obstructive individuals (n = 22) (p = 0.040).

UPF17 had a stronger PARP-1 mRNA expression level lowering effect amongst patients with COPD, as compared to that amongst non-obstructive individuals (non-obstructive non-smokers and non-obstructive smoking individuals) (p = 0.040) (figure 3).

## Discussion

After adjustment for demographic variables, BMI, smoking-related indices and lung function, in the PBMC, the common Comet Assay IV parameters indicative of DNA damage, such as tail length, tail migration, olive tail moment and DNA % in the tail, showed a significant positive dependence on progression throughout the scale from healthy non-smoking controls to severe exacerbations of COPD. Formerly, the issue of stage- or progression-dependence of the DNA damage in COPD has remained controversial. Ceylan et al. showed an increased DNA damage in peripheral blood mononuclear leukocytes in patients with COPD [1], but using the same technique, Maluf et al. did not detect a correlation between the increased DNA damage in PBMC and COPD severity [18]. The major difference between the current study and those conducted by Ceylan et al. and Maluf et al. was that in the current one, a special software (Comet Assay IV) was used to analyse at least 10 different variables of the comet assay instead of visual scoring of the tail intensity only. Furthermore, in our study 7 groups were analysed using ordinal logistic regression to test for the progressing DNA injury from non-smoking control and non-obstructive smokers through the four COPD stages to severe COPD exacerbation. The DNA damage along with PARP activity and PARP-1 mRNA expression has not been investigated formerly in this way. *In-vitro* studies have shown relations of PARP activity and cigarette smoke [19], [20]. Also, differential activation of PARP in PBMC from controls and COPD patients has been shown previously [7], but *in-vivo* studies on the relationships between PARP activity and COPD progression have not been performed before. Not less importantly, all variables indicative of DNA damage were adjusted for parameters like age, gender, BMI, smoking habits and lung function. In the context that COPD is a disease of systemic senescence [21], the results adjusted for the demographic and clinical parameters point out that the increase in DNA damage is likely to increase accordingly to the advancement of the OS and inflammation from non-smoking controls up to COPD exacerbation, rather than caused by age, by fall in FEV_1_% or by cigarette smoke (CM) alone. Adjustment to gender might be of particular importance, as the PBMC PARP activity has been shown to be significantly lower amongst female subjects compared with male subjects [22]. Moreover, according to rodent [23] and porcine [24] models, the regulation of PARP has been reported to be more profound in males than in females. In addition to the variables provided by the Comet Assay IV, 5 new parameters were introduced and found to reflect the relationship between DNA damage and the progression of the disease even better. Out of the novel or newly introduced parameters, the tail length/cell length ratio and tail migration/cell length ratio were found to show the highest ORs with the development and progression of COPD up to COPD exacerbation. A one unit increase in tail length/cell length ratio and tail migration/cell length ratio was associated with a 7.88-fold or 3.91-fold higher probability, respectively, for the process progressing to the next stage and therefore can appear most suitable to describe the DNA damage in PBMC over the evolution of COPD. This is the first report to show that these new parameters are most sensitive for the advancement of COPD and to suggest the tail length/cell length ratio and the tail migration/cell length ratio as new precision tools for independent assessment of the DNA damage in smokers and in patients with COPD.

Along with the comet assay indices, the current study revealed a strong relationship between the evolution of COPD and PARP activity in PBMC. These data suggest an intensified DNA repair occurring in PBMC of patients with higher COPD stages and adds a valuable adjunct to understanding of the systemic component of COPD. PARP activity was somehow heightened amongst the non-obstructive smokers, as compared to both the non-smoking controls on one hand and to patients with mild COPD on the other (table 4, figure 2). This may indicate that those smokers, who do not develop COPD, are still sensitive to various antioxidant influences than do COPD patients, at least those with a milder disease. This suggests that although PARP is a very important player in the DNA repair, it does not represent the only pathway to prevent smokers from developing COPD. In mammalian cells, the basal enzymatic activity of PARP is very low, but oxidative and nitrosative stress can trigger DNA strand breakage, which in turn abruptly stimulates the PARP-1 activity [20], [25]–[27]. Moderate activation of PARP is of physiological importance and could be beneficial via facilitating the DNA repair. However, over-activation of PARP is connected to numerous pathological conditions associated with oxidative and nitrosative stress, including COPD [28]. In particular, in parallel with energy depletion by the cells caused by PARP over-activation, PARP contributes to the synthesis of inflammatory mediators including cytokines, chemokines and enzymes like inducible nitric oxide synthase, but also up-regulates expression of pro-inflammatory genes, which leads to augmented inflammation [6]. Thus, the DNA damage caused by chronic OS as one of the main mechanisms of COPD can activate PARP. As a consequence, systemic PARP over-activation may play an important role by up-regulating the expression of pro-inflammatory genes in COPD [29], [30] leading to a vicious circle of consecutively increased OS and inflammation. In this light, one could speculate that the over-activation of PARP can be one of the intermediate mechanisms for the abnormally augmented inflammation in COPD [31]. If true, inhibition of PARP activity could provide protection against OS-related inflammation and thereby afford therapeutic benefits in COPD [32], [33].

Our study showed that UPF17, a non-toxic and ∼3000-fold better hydroxyl radical scavenger than GSH [34], [35], has a significant inhibitory effect on PARP-1 mRNA expression in patients with COPD, compared to that in non-obstructive individuals. Our latest experiments on HepG2 cells suggested that the activity of UPF17 is mediated via the nuclear factor (erythroid-derived 2)-like 2 (Nrf2) (unpublished data). Although, inhibiting PARP can have anti-inflammatory effects, inhibiting PARP in a greater degree reduces DNA repair in cells [36], which can be considered as a side effect in the context of COPD. Collectively, the clinical significance of PARP-1 down-regulation remains elusive.

Prospective nature, inclusion of people from healthy controls through non-obstructive smokers to severe exacerbation of COPD into ordinary logistic regression models adjusted for major demographic, clinical and smoking-related parameters can be considered as strengths of the study. Small and unequal study groups and different populations for comet assay and assessment of PARP-1 mRNA expression are clear limitations.

Taken together, the present results show that COPD is a systemic disease and that DNA damage and PARP activation in PBMC are related both to the progression of COPD, but also to its exacerbation. The newly derived comet assay parameters (tail length/cell length ratio and tail migration/cell length ratio) can better reflect the changes occurring along with the evolution of COPD than the standard parameters that were used up to now. Furthermore, UPF17 was effective in modulating the expression of PARP-1 mRNA. However, it remains to be elucidated whether a modest inhibition of PARP-1 may have a safe enough potential to break the vicious cycle of the development and progression of COPD.
